# Anti-Hu antibody associated paraneoplastic neurological syndrome in a child with ganglioneuroblastoma: A rare case report and literature review

**DOI:** 10.1097/MD.0000000000038148

**Published:** 2024-05-10

**Authors:** Yi-Ling Dai, Ling Xiao, Zhen Pan, Guo-Qian He, Ju Gao, Xia Guo, Zhuo Huang

**Affiliations:** a Key Laboratory of Birth Defects and Related Diseases of Women and Children, Ministry of Education, West China Second University Hospital, Sichuan University, Chengdu, Sichuan, P.R. China; b Department of Pediatrics, West China Second University Hospital, Sichuan University, Chengdu, Sichuan, P.R. China; c Sichuan University, Chengdu, Sichuan, P.R. China.

**Keywords:** anti-Hu, antineuronal nuclear antibodies (ANNA), child, ganglioneuroblastoma, paraneoplastic neurological syndrome

## Abstract

**Rationale:**

Paraneoplastic neurological syndrome with anti-Hu antibody (Hu-PNS) is a neurological disorder that occur in patients with malignancy. The syndrome has a wide range of presentations and can present before diagnosis of primary malignancy. Familiarity with these paraneoplastic neurological syndromes can help early recognition and take appropriate regimens.

**Patients concerns:**

Diagnosis and treatment of Hu-PNS.

**Diagnoses:**

This is retrospective study that analyzed the clinical data of this case. Through retrospective analysis and targeted antibody screening, serum anti-Hu antibody was detected. Subsequent spinal imaging revealed a mass in the paraspinal region, which was confirmed as ganglioneuroblastoma by pathologic examination.

**Interventions:**

The child was treated with a course of intravenous immunoglobulin and radical surgical operation without chemotherapy.

**Outcomes:**

The neurological symptoms were gradually improved and no signs indicate disease progression or tumor recurrence.

**Lessons:**

Hu-PNS has rarely been reported in children with ganglioneuroblastomas. They can mimic non-neoplastic processes, making detection and diagnosis difficult. Serum and/or cerebrospinal fluid onconeural antibody can strongly indicate occult cancers. Early detection of paraneoplastic neurological syndromes can help take appropriate regimens and improve prognosis.

## 1. Introduction

Paraneoplastic neurological syndrome (PNS) describes a misdirected immune response against neural proteins due to systemic cancers that cause irreversible neuronal loss and severe neurological disability.^[1]^ It affects either the central or peripheral nervous system and is characterized by various clinical syndromes.^[2–4]^ The symptoms and signs are diverse including ataxia, myoclonus, opsoclonus, and complex partial seizures, which are often misdiagnosed as other nervous system diseases.^[5]^ And it is difficult to diagnosis due to underlying tumors are often asymptomatic and sometimes occult.^[6,7]^ Familiarity with these neurological syndromes can aid in early recognition and approximate treatment.^[8]^

PNS is associated with a variety of onconeural antibodies and it can be divided into 3 categories according to target localization: disorders associated with antibodies against intracellular antigens, disorders associated with antibodies against intracellular synaptic epitopes, and disorders in which the antigens are located on the cell surface or are synaptic proteins.^[7,9,10]^ Anti-Hu antibody, also known as the type I antineuronal nuclear antibody-1, can specifically bind to the nuclear antigens.^[11,12]^ It most likely induces a T cell-mediated immune response rather than directly against intracellular antigens.^[13–15]^ PNS with anti-Hu antibody (Hu-PNS) refers to a spectrum of severely disabling disorders that can be detected in various types of cancer, most commonly small cell lung cancer.^[16,17]^ It has rarely been reported in children and was primarily found in patients with neuroblastic tumor, which arise from the sympathoadrenal lineage of the neural crest.^[18]^ Based on the morphology, clinical features, and biological behavior according to the International Neuroblastoma Pathology Classification, neuroblastic tumor can be divided into 4 categories: neuroblastoma, nodular ganglioneuroblastoma, intermixed ganglioneuroblastoma, and ganglioneuroma.^[19]^ Ganglioneuroblastoma has cell polymorphisms in ganglionic cells with different degrees of maturation and calcification. The degree of malignancy is intermediate between that of neuroblastomas and ganglioneuromas.^[20,21]^ At present, a few ganglioneuroblastoma-associated Hu-PNS cases have been reported^[22–24]^ and we addressed anti-Hu-associated topics by describing a rare case with ganglioneuroblastoma.

## 2. Case description

A 2-year-old boy was admitted to West China Second University Hospital presented with gait disturbance for more than 2 months. During this period, the symptoms could resolve spontaneously. However, the symptoms recurred after a respiratory viral infection. In the following days, the gait disturbance became much more severe and left eye esotropia appeared progressively, especially when looking at or following certain objects. His personal and familial medical histories were unremarkable and he did not expose to any drugs or poisons. Physical examination revealed cerebellar ataxia and left eye esotropia. He was too young to cooperate with the finger-to-nose test, heel-knee-shin test, rapid alternating movement test and Romberg tests. No blepharoptosis, nystagmus or ophthalmoparesis was observed. Convergent eye movements were normal and there was no evidence of facial palsy. The cognitive function, muscle force and muscle tension were normal.

Ataxia is a fairly common neurologic symptom and this could be the foundation of the investigation of its etiology. Ataxia is a non-localizing complaint and it is most frequently caused by dysfunction of the complex circuitry connecting the basal ganglia, cerebellum, and cerebral cortex or dysfunction of proprioceptive sensory activity. According to onset form, ataxia can be divided into acute, subacute, chronic, or intermittent. The child had an acute onset and the symptoms evolved rapidly over weeks. The causes of acute ataxia in childhood mainly include infection or post-infectious autoimmune reaction, paraneoplastic syndrome, vascular factors, poisoning, trauma, metabolic disease, and psychogenic impairment. Our patient was 2 years old and did not have a history of trauma or poisoning. We ruled out poison, trauma or psychogenic impairment. Considering that his symptoms could resolve spontaneously and reemerged after respiratory viral infection, it was unlikely to be related to vascular factors or metabolic diseases. Hence, infection, post-infectious autoimmune reactions and paraneoplastic syndrome were highly suspected and we performed targeted assistant examinations to validate it.

**Figure 1.**
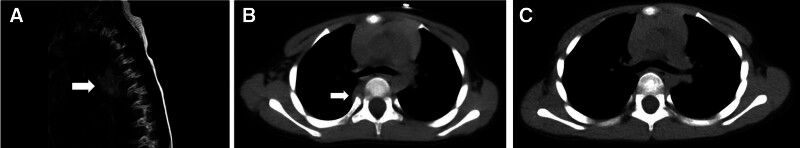
Imaging of the paraspinal region revealed cancer. Spinal MRI (A, white arrows) and chest CT (B, white arrows) showed an irregular soft tissue mass at the T5 to T6 vertebral levels that were adjacent to the bronchus principal dexter, which was enhanced with contrast administration. (C) Chest CT showed no irregular soft tissue mass. CT = computed tomography, MRI = magnetic resonance imaging.

The blood test showed no abnormalities, including routine blood tests, C -reactive protein, biochemical tests, thyroid function tests, metabolic screening and autoantibodies (nRNP/Sm, Scl-70, ANA, dsDNA, Smith, SS-A, SS-B, Jo-1, PM-Scl, CENPB, PNCA, NU, HI, RIB, and M2). Brain magnetic resonance imaging (MRI) revealed no lesions or inflammation in the cerebellar hemisphere. Cerebrospinal fluid examination revealed leukocytosis (188 × 10^6^/L) with 93% lymphocytes and a slightly elevated protein level (1601.5 mg/L). The serum and cerebrospinal fluid were assessed simultaneously for IgG index, 24 hours intrathecal IgG synthesis rate, oligoclonal bands and antibodies against neuronal cell surface (NMDAR, AMPAR, GABA_B_R, LGI1, GAD65, and Caspr2). The results of oligoclonal bands and antibodies against the neuronal cell surface were negative, but the IgG index and 24 hours intrathecal IgG synthesis rate were slightly elevated. Serum anti-Hu antibody were detected (titer 1:10) by western blotting (V-Medical Laboratory Hangzhou China) but cerebrospinal fluid anti-Hu antibody was not assessed due to insufficient sample size. No tumor cell was detected on microscopic examination of cerebrospinal fluid. Although the antibody titer was low, anti-Hu antibody is a well-characterized onconeural antibody and further examinations were performed. Spinal MRI showed an enhancing 2.5 cm × 0.8 cm lesion in the paraspinal on the thoracic 5 to 6 region (Fig. 1A). Chest computed tomography (CT) also confirmed this finding (Fig. 1B) and abdomen and pelvis CT showed normal findings.

Written informed consent was obtained from the legal guardian for treatment and the child was empirically treated with a course of intravenous immunoglobulins (2 g/kg). After treatment the ataxia and left eye esotropia were gradually relieved and radical tumor resection was performed 14 days later (calculated from the start of intravenous immunoglobulins). Histopathology identified the tumor as ganglioneuroblastoma without C-Myc gene or N-Myc gene amplification. He did not receive postoperative chemotherapy and immunosuppressive treatment. During the follow-up, the neurological symptoms completely disappeared without tumor recurred. The anti-Hu antibody was continuously negative and spinal CT was performed per 6 months and showed no abnormality (Fig. 1C).

## 3. Discussion

PNS is a group of immune-mediated neurological syndromes associated with the remote effects of tumor instead of the infiltrative growth or metastases.^[25]^ The diseases have an acute or subacute onset with transient spontaneous remission and virus infection can be possible precipitating factor.^[26]^ The diagnostic clues can refer to the following points: any parts of the nervous system can be affected; immune-mediated pathogenesis, which is confirmed by the presence of specific neuronal antibodies; and the underlying tumors can be detected.^[27]^ Hu-PNS is the most frequent PNS associated with well-characterized onconeural antibody. It is a spectrum of severely disabling disorders that can affect the brainstem and cerebellum, including the dorsal root ganglia and the peripheral nerves. The prognosis for adult Hu-PNS is generally poor and more than half of the patients have been reported to become bed- or wheelchair-bound, only 5% to 7% of patients can relive.^[28–31]^ Patients with Hu-PNS will have a better prognosis if the syndromes are recognized timely and tumor is found in the early stage. Familiarity with these paraneoplastic neurological syndromes can help accelerate the diagnostic process, take timely interventions and reduce tumor mortality.

Anti-Hu antibody is a “well-characterized onconeural antibody” and specifically bind to the nuclear antigens of both peripheral and central nervous system neurons.^[11]^ The term “well-characterized antibody” is based upon the following points: The antibodies can be recognized on routine immunohistochemistry and can be confirmed their specificities on recombinant proteins; There have been a number of case reports associated with tumors; The description of well-characterized neurological syndromes associated with the antibodies; The unambiguous identification of the antibodies among different studies.^[32]^ Anti-Hu antibody is definitely associated with several occult and progressive tumor and can exhibit multifocal neurological symptoms. If a patient was positive for this antibody, the probability of tumor was >95%.^[33,34]^ In adults, PNS is found in approximately 75% of patients with small lung cell cancers but in children, it is primarily found in pediatric neuroblastic tumor.^[8,18]^ Ganglioneuroblastoma is a peripheral neuroblastic tumor, derived from neuronal cells of the sympathetic nervous system and represents 20% of all neuroblastic tumors.^[35]^ It can be found anywhere in the sympathetic system and is usually unilateral and does not cross the midline. The manifestation depends on tumor position, pathological grade and stage. At the early stage, the clinical manifestations are nonspecific, including pain, fever, marasmus. It rarely manifests as chronic diarrhea due to the increased production of vasoactive intestinal peptides.^[36]^ Giant mediastinal tumor may present with dyspnea and superior vena cava syndrome. The therapeutic regimen of ganglioneuroblastoma-associated Hu-PNS includes tumor resection, immunotherapy and symptom control. Immunotherapy can alleviate neurological symptoms and radical surgery can aid eradicate tumor and clarify diagnosis. Dissimilar immunosuppressive treatments have been used for Hu-PNS, including steroids, intravenous immunoglobulin, immunosuppressants (cyclophosphamide and rituximab) and plasma exchange.^[37]^ Patients with onconeural antibodies against surface or synaptic antigens are more likely to have a marked improvement compared to patients with antibodies against intracellular antigens.^[32]^ Currently, some clinical trials have used programmed cell death protein 1 inhibitors to treat Hu-PNS, but the results are disappointing. The programmed cell death protein 1 inhibitors can ameliorate the disease course but no superior effect to those of other reported immunosuppressive treatments were observed.^[38,39]^ The malignant potential of ganglioneuroblastoma lies between that of neuroblastomas and ganglioneuromas.^[19]^ Chemotherapy should be considered based on the pathological subtype.

**Figure 2.**
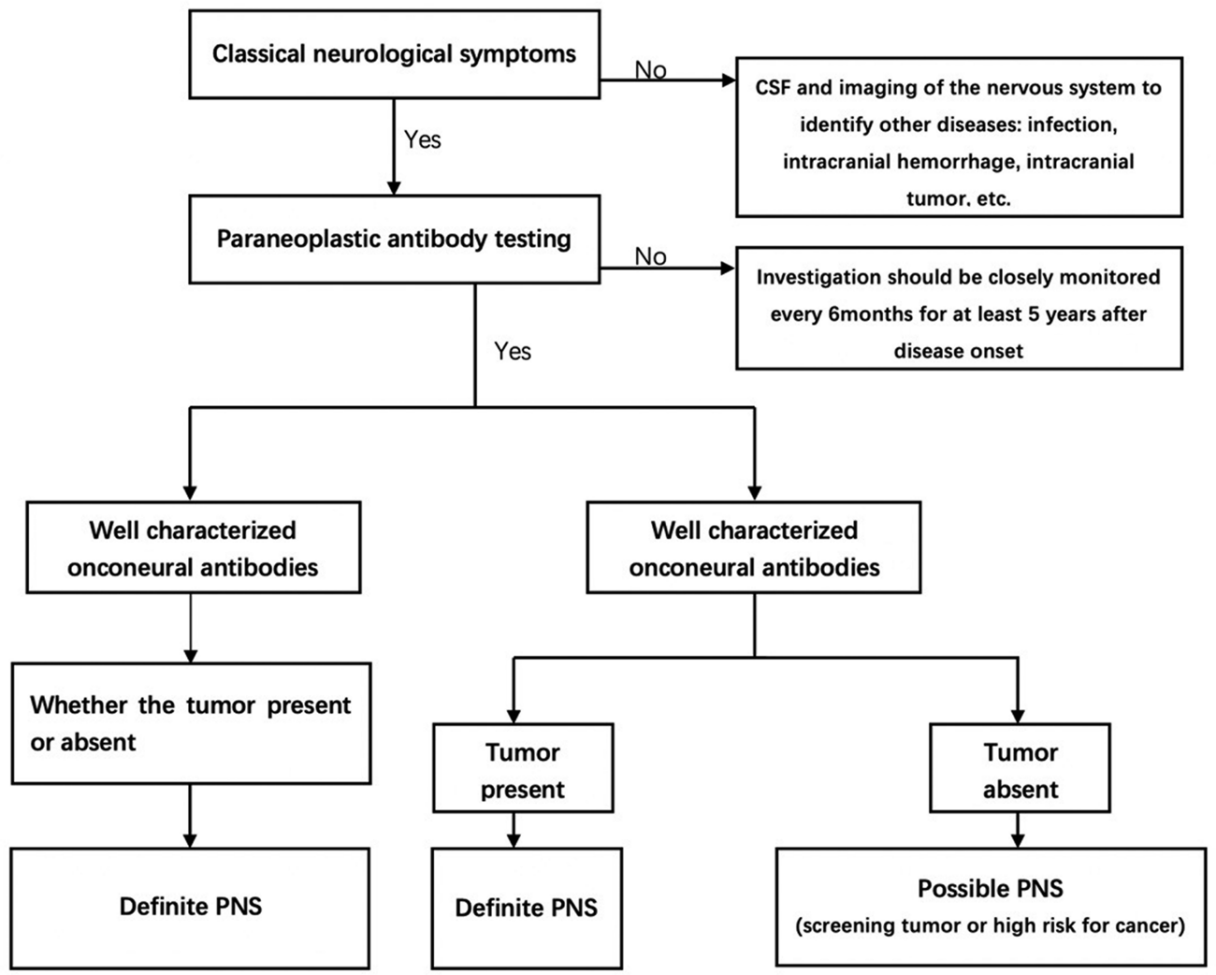
Flow chart showing the level of diagnostic evidence of the paraneoplastic neurological syndrome.

In our case, the child was treated with intravenous immunoglobulin and radical surgery. No regional or remote metastasis was detected and C-Myc gene or N-Myc gene was not amplified; therefore, chemotherapy was not performed after careful discussion. Considering the anti-Hu antibody titer in the serum was low (1:10) and the symptoms were gradually relieved after immunoglobulin infusion, the child was not given another intensive immunosuppressive treatment. During the follow-up, his symptoms completely disappeared, anti-Hu antibody was continuously negative and postoperative routine spinal CT did not discover any abnormalities. We proposed the process of disease management for PNS in Figure 2. Tumor resection is the fundamental treatment option. Not all tumors require chemotherapy and not all Hu-PNS patients require aggressive immunotherapy. But our limitation is that we did not calibrate anti-Hu antibody titer in cerebrospinal fluid and the relationship between anti-Hu antibody and the neurological syndrome has not been further confirmed.

## 4. Conclusion

Hu-PNS has rarely been reported in children with ganglioneuroblastomas. The symptoms and signs of PNS can precede the appearance of tumor and disease may be misdiagnosed. Serum and/or cerebrospinal fluid onconeural antibody can strongly indicate occult cancers. Familiarity with these paraneoplastic neurological syndromes can help early recognition and make appropriate regimens. Treatment with Hu-PNS includes tumor resection, immunotherapy and symptom control. The therapeutic regimen should base on clinical manifestations and tumor types and not all patients require intensive treatment.

## Acknowledgments

The authors thank the patient parents for providing permission to use their children information.

## Author contributions

**Conceptualization:** Ju Gao.

**Data curation:** Ling Xiao.

**Formal analysis:** Xia Guo.

**Funding acquisition:** Guoqian He.

**Investigation:** Guoqian He, Zhuo Huang.

**Resources:** Zhen Pan.

**Supervision:** Ju Gao.

**Validation:** Xia Guo.

**Writing – original draft:** Yi-Ling Dai.

**Writing – review & editing:** Zhuo Huang.
